# Spinal dural arteriovenous fistulas: a report on outcomes in a single-center retrospective cohort treated surgically and/or endovascularly

**DOI:** 10.3325/cmj.2021.62.347

**Published:** 2021-08

**Authors:** Miroslav Vukić, Hrvoje Barić, David Ozretić, Ivan Jovanović, Zdravka Poljaković, Katarina Tudor, Krešimir Saša Đurić

**Affiliations:** 1 Department of Neurosurgery, University Hospital Center Zagreb, Zagreb, Croatia; 2 Zagreb University School of Medicine, Zagreb, Croatia; 3 Croatian Institute for Brain Research, Zagreb University School of Medicine, Zagreb, Croatia; 4 Department of Neuroradiology, University Hospital Center Zagreb, Zagreb, Croatia; 5 Department of Neurology, University Hospital Center Zagreb, Zagreb, Croatia

## Abstract

**Aim:**

To report on the outcomes of spinal dural arteriovenous fistulas (sDAVFs) treatment in a single-center retrospective cohort.

**Methods:**

Data were retrieved on sDAVF cases treated surgically and endovascularly between January 2009 and January 2020. Sociodemographic, clinical, imaging data, and outcomes were analyzed.

**Results:**

Thirty-four patients were identified: 11 female, mean age 64.1 ± 11.5 years; mean time of symptom duration 12 (range 1-149) months. The sDAVF locations were the following: 18 (62.1%) thoracic, 4 (13.8%) lumbar, 4 (13.8%) sacral, and 3 (10%) with multiple location feeders. All patients had a motor deficit and affected walking, and the majority had a sensory deficit, bowel, and bladder dysfunction. Fifteen (44.1%) patients underwent surgical treatment, 7 (20.6%) underwent endovascular treatment, and 12 (35.3%) underwent both (crossover). Radiological myelopathy showed regression in 19 (55.9%) patients. Overall, clinical improvement (decrease in modified Rankin score) following treatment was observed in 14 patients (41.2%), worsening in 1 (2.9%), while other had unchanged status. The proportion of patients with initial treatment failure markedly differed between the before-2014 and after-2014 period. Patients who failed to improve had more extensive myelopathy.

**Conclusion:**

Patients who underwent surgery or endovascular treatment had on average significant clinical recovery, while those who underwent treatment crossover had negligible improvement. The extent of myelopathy seems to be associated with clinical improvement.

Spinal dural arteriovenous fistulas (sDAVF) are rare vascular malformations, with an estimated annual detection rate of 0.29/100 000 and probably a much higher incidence; however, among the spinal vascular lesions they are the most common entity (1,2). They pose a significant challenge both diagnostically and therapeutically due to an insidious nonspecific presentation resulting in a misdiagnosis or delayed diagnosis and high disability rates (3). The sDAVF is an arteriovenous shunt fed by a radicular artery and drained by a medullary vein of the coronal venous plexus (4). The arterialized blood retrogradely fills the venous plexus leading to congestion and venous congestion, ultimately resulting in spinal cord edema, hypoperfusion, and ischemia (5). This pathophysiological sequence manifests as the clinical spectrum of myelopathy: from chronic gait disturbances and motor and sensory lower extremity deficits to the Foix-Alajouanine syndrome, a subacute necrotic myelopathy (6).

Treatment options for the majority of sDAVFs include open surgical resection and endovascular treatment, with a minority of cases being treated conservatively and radiosurgically (6). There is a lack of high-quality clinical trial-based evidence to unequivocally answer the question on optimal treatment. Cohort data seem to suggest the superiority of open surgery, but other modalities have their advantages and are continued to be used as individual anatomy and other factors affect the choice of treatment (4,5,7,8).

This study aims to report on a series of sDAVF cases treated over a 10-year period at our Institution using endovascular, surgical, or a combination of treatments. Clinical and radiological outcomes were analyzed and compared across treatments, and outcome predictors were investigated.

## METHODS

The study was approved by the Institutional Review Board of University Hospital Center Zagreb. The requirement of obtaining patient consent was waived by the Institutional Review Board.

### Study design and setting

Institutional patient archive (digital and hard-copy patient charts) was retrospectively reviewed for all sDAVF cases treated at University Hospital Center Zagreb, Departments of Neurology, Radiology, and Neurosurgery, between January 2009 and January 2020. Patients treated using open surgery, endovascular treatment, or a combination of both (treatment crossover in case of initial failure) were included in further analyses.

### Participants, variables, and data sources

We included adult patients with a radiologically confirmed sDAVF: myelopathy on magnetic resonance imaging (MR) and an arteriovenous fistula proven on digital subtraction angiography (DSA) or MR-angiography (MRA). The exclusion criteria were age <18 years and exclusively conservative treatment.

The following data were collected: i) demographics (age, sex); ii) duration of symptoms; iii) modified Rankin score (mRS) before and after treatment; iv) type of clinical symptoms (motoric deficit, sensory deficit, gait disturbance, and bladder and bowel dysfunction); v) comorbidities; vi) pre- and postoperative MR findings (level and extent of myelopathy, presence of an engorged venous plexus, level of fistula, residual disease, recurrent disease); vii) pre- and postoperative DSA findings (type and level of fistula, residual disease); viii) treatment (type and number of procedures).

Symptom duration was defined as the time from symptom onset to procedure; myelopathy was defined as T2 intramedullary hyperintensity; fistulas were defined as being cervical, thoracic, lumbar, sacral, or mixed if there were multiple feeders across adjacent segments. Other than the anatomical classification, no further sDAVF subdivisions were used. Patients were followed-up radiologically (MR/MRA angiography) before discharge and then at three to six months post-treatment. Patients who failed to improve after endovascular treatment underwent a follow-up DSA after a relapse was noted. Clinical follow-up was at three months, six months, and then once annually. Clinical symptoms were not graded, rather they were coded as a binary variable. As per indication, workup was scheduled earlier. Clinical status was assessed at admission, and postoperatively at the three-month follow-up. The most recent follow-up clinical and radiological data were analyzed. All surgical procedures were carried out by a senior neurosurgeon (MV); endovascular procedures (diagnostic and therapeutic – Onyx^®^ was used in two endovascular procedures, N-butyl cyanoacrylate in the remaining cases) and MR assessments were performed by radiologists, authors of this study; clinical assessments were performed by neurologists – both radiological and clinical assessment were independently performed by two investigators, then findings were compared and any inconsistencies resolved by consensus. Data that were not explicitly stated were interpreted from the patient's record (eg, if mRS was not entered in the discharge letter, it was calculated independently by the two neurologists using data from the discharge letter). Perioperative care, and surgical and endovascular procedures, were all performed in a standard manner, as reported in the literature (5,9).

### Data analysis

Continuous variables were summarized as median (range) and categorical variables as absolute (relative) frequencies. For outcome analysis, patients were grouped according to pre- to post-treatment mRS difference into: a) improvement group (mRS difference of 1 or higher); b) no improvement group (no change or increase in mRS). Effects were expressed as median difference for continuous variables, and unadjusted odds ratios (95%) for nominal variables. The nominal variable “type of treatment” was dichotomized into “single” (endovascular treatment or surgery) vs “combined” and the variable “number of treatments” into “single” (one procedure) vs “more” (two or more procedures). Pre- to post-treatment mRS differences were compared across treatment groups (endovascular, surgical, and crossover). Finally, the number of individual procedures was compared across five-year periods (early: 2009-2014, and late: 2015-2019), since the cutoff roughly coincides with systemic organizational changes in the national management protocol of sDAVFs. Namely, up to 2014, patients were in general initially diagnosed and treated endovascularly in different centers, and only sent to surgery after a failed endovascular procedure.

## RESULTS

Thirty-four patients were confirmed eligible and included in the study: 15 in the surgical group (S); 7 in the endovascular group (E); and 12 in the treatment crossover group. Clinical follow-up was available for 32 (94.1%) patients, radiological follow-up for 24 (70.6%). Individual patient data are shown in Table 1. Overall, there were 11 female patients; median age 65.5 years; median time of symptom duration was 12 months (range 1-149). The most common sDAVF location was thoracic (n = 8, 62.1%), 3 (10.3%) had multiple location feeders. All patients had a motor deficit and all had affected walking; sensation loss occurred in 29 (85.3%) patients; the majority of patients had impaired bowel/bladder function (Table 2).

**Table 1:** Individual patient characteristics*

Patient number^†^	Sex		Age		Time to diagnosis (months)	Level	Treatment^‡^	Myelopathy segments	Follow-up imaging	Modified Rankin score	
										pre	post
1	F	72		8		Th10	E	Th5-conus	None	5	-
2	M	57		1		Th6, Th7	S, S, E	Th6-conus	DSA – fistula obliterated	4	4
3	M	63		24		Th12, L1, L2	S, E	Th7-conus	MR/MRA/DSA – minimal myelopathy, fistula obliterated	4	4
4	F	78		17		L1	S, E	Th5-conus	MR/MRA – myelopathy regression, fistula obliterated	4	4
5	F	53		18		S1	E	Th8-conus	None	3	1
6	F	72		5		Th12, L1	E, E	Th10-conus	MR/DSA – myelopathy, suspected fistula	2	1
7	M	62		-		sacral	E	Th6-conus	MR/DSA – no myelopathy, fistula obliterated	4	1
8	M	61		39		Th6	S, E, E	Th6-conus	MR/DSA – myelopathy regression, fistula obliterated	5	5
9	M	62		7		Th5	E	Th3-conus	None	5	-
10	M	56		37		Th10	S, E, E	Th9-conus	MR/MRA – no myelopathy, fistula obliterated	5	4
11	F	68		6		Th6	S, E	Th8-conus	MR/MRA – no myelopathy, fistula obliterated	1	0
12	F	66		105		Th6	S	Th6-Th10	MR/MRA – myelopathy regression, fistula obliterated	5	4
13	F	76		12		Th8	S, E	Th8-conus	MR/MRA – myelopathy, fistula obliterated	4	4
14	M	69		149		Th6, Th7	S, S, E	Th6-Th7	MR/MRA – no myelopathy, fistula obliterated	2	1
15	M	54		1		Th7	S	Th6-conus	None	5	4
16	M	73		11		S1	S	Th4-conus	DSA – fistula obliterated	5	5
17	M	75		12		Th7, Th9	S	Th7-conus	MR/MRA – myelopathy regression, suspected fistula	4	4
18	M	73		8		L1	S	-	None	1	0
19	F	74		15		L2	S, S	Th3-conus	None	3	2
20	M	74		12		Th7	S	Th7-conus	None	4	4
21	F	77		15		Th11	S, S	Th6-conus	None	5	5
22	M	58		96		Th7	S	Th3-conus	None	4	4
23	M	73		13		Th9	E	-	MR/MRA/DSA – no myelopathy, fistula obliterated	3	2
24	F	63		17		S1	S, E	Th3-conus	MR/MRA – myelopathy regression, fistula obliterated	5	5
25	M	67		2		L4	S, S	Th5-conus	MR/MRA – no myelopathy, fistula obliterated	5	5
26	M	65		2		V4	E, E, S	C1-Th2	MR – myelopathy regression	5	4
27	M	33		1		-	E	-	None	5	5
28	M	56		1		-	E, E, S	-	None	4	5
29	M	67		26		Th5	S	Th5-conus	MR – myelopathy regression	5	4
30	F	72		1		-	S	-	MRA/DSA – fistula obliterated	0	0
31	M	58		1		-	S	-	MR/MRA – no myelopathy, fistula obliterated	0	0
32	M	64		56		Th9	S	Th9-L1	MR – myelopathy regression	4	3
33	M	24		9		Th7	S, S	Th5-Th11	MR – myelopathy regression	3	2
34	M	64		120		Th4	E, S, S	Th4-conus	MR – myelopathy regression	4	4

*Abbreviations: procedure: S – surgery, E – endovascular; pre – preoperative; post – postoperative (latest follow-up); MR – magnetic resonance; MRA – magnetic resonance angiography; DSA – digital subtraction angiography; V4 – 4th segment of the vertebral artery.

†patients 1-14 were treated before 2014, patients 15-34 after 2014.

‡treatments (E, S) are listed chronologically.

**Table 2:** Preoperative patient data summary*

Variable	n/median	%/range
**Sex (F/M)**	11/23	32.4/67.6
**Age (years)**	65.5	24-78
**Duration of symptoms (months)**	12	1-149
**Level of fistula**		
thoracic	18	62.1
lumbar	4	13.8
sacral	4	13.8
multiple location feeders	3	10.3
**Motor deficit (paraparesis)**		
spastic	19	55.9
flaccid	8	23.5
transitory	3	8.8
normal reflexes	4	11.8
**Sensory deficit**		
yes	29	85.3
no	5	14.7
**Ambulation**		
unable to walk	14	43.8
uses an aid	10	31.3
independent	8	25
**Bowel function**		
constipation	17	50
incontinence	4	11.8
normal function	8	23.5
unknown	5	14.7
**Bladder function**		
incontinence	13	38.2
retention	14	41.2
lower urinary tract symptoms	4	11.8
normal function	3	8.8
**Modified Rankin Score**	4	0-5
**Treatment overall**		
surgery	15	44.1
embolization	7	20.6
crossover treatment	12	35.3
**Treatment before 2014**		
surgery	4	23.5
embolization	5	29.4
crossover treatment	8	47.1
**Treatment after 2014**		
surgery	11	64.7
embolization	2	11.8
crossover treatment	4	23.5

*Numbers are median (range), or absolute (relative) frequencies.

Of the 24 patients who underwent postoperative radiological follow-up, 2 patients had a suspected residual fistula; myelopathy regression was seen in 17 (70.8%) patients. Fifteen patients underwent a single active treatment (either surgery or endovascular): 8 experienced improvement in mRS, the rest had no change in mRS. Among the 17 crossover patients, 7 had mRS improvement, one experienced mRS deterioration, and others experienced no change in mRS. The proportion of crossover cases (ie, initial treatment failure) markedly differed between the before-2014 and after-2014 period: 8 (47.1%) vs 4 (23.5%). Median time-to-diagnosis was comparable between the time periods: 12 (range 1-149) months vs 12 (range1-120) months.

## DISCUSSION

In the present study, we reported and compared clinical and radiological findings and outcomes in patients with sDAVF who underwent surgery, endovascular treatment, or a combination of both. The data support the role of open surgery as the first-line treatment.

There were no unexpected findings in our cohort with regard to sociodemographic characteristics, presentation, and morphological features of the lesion. The majority of our patients were men, on average in their seventh decade, and the majority of the lesions were in the thoracic region (10).

The overall clinical improvement rate of 46.9% is comparable with previous reports: Jablawi et al (11) reported clinical improvement in 53% of 40 patients treated by microsurgery; Tsuruta et al (12) reported improvement in 48.3% of 172 sDAVF cases treated by endovascular treatment. However, comparing clinical outcomes across published data on sDAVF is difficult, since the majority of studies report angiographic findings as primary outcomes, and those reporting clinical outcomes lack consistency in assessment scales as both mRS and the Aminoff Logue Scale are used. In their meta-analysis from 2019, Goyal et al (7) identified 32 studies comparing endovascular and surgical treatment, only 13 of which reported clinical outcomes. Angiographic improvement (obliteration of fistula) was high in our cohort (91.7%) – a rate comparable to previously published results (11-13). Our results show that patients undergoing either surgery or endovascular treatment as the single treatment modality experience better clinical improvement than patients undergoing both treatments, who fail to improve. This finding on the lack improvement in patients undergoing both treatments is consistent with previously published results and is expected since the need for additional treatment implies poor initial treatment response and comparably more complex cases (9). We were not able to meaningfully compare the two treatments employed as a sole strategy due to a small sample size. Data available in the literature, including the most recent meta-analysis on the subject, show the superiority of surgical treatment in terms of clinical outcomes (7). Studies also show the superiority of endovascular treatment, but they were burdened by imprecise estimates due to small sample size and yielded inconclusive results (14-16).

We compared the incidence of the treatment type across two five-year periods – “early” (2009-2014) and “late” (2015-2019). A nationwide consensus was reached in 2014 to start referring all sDAVF patients to our center and consider surgery as the first-line treatment owing to growing evidence on the superiority of open surgery. The shift in policy is evident from our data – in the second period, the time to diagnosis decreased, the number of surgical procedures more than doubled, the number of endovascular procedures decreased more than two times, and, most importantly, the number of treatment crossover cases decreased two times. Starting from 2015, only two sDAVF patients have undergone endovascular treatment at our Institution as the primary treatment strategy – three patients with initial endovascular failure were referred to us after unsuccessful (one or two) endovascular treatments at another center. These are important results based on which we expect even better clinical outcomes in our future sDAVF cases. In addition, we noticed an “experience curve effect” in our treatment strategy with regard to both diagnosis and treatment. For example, there were two cases of wrong-level diagnosis among the “early group” patients, while no such complications were noted among patients in the more recent group.

Our analysis showed that the extent of myelopathy might be associated with the outcome – patients who failed to clinically improve had more extensive myelopathy on the preoperative MRI. More extensive myelopathy might also correlate with clinical severity (due to longer duration of fistula before presentation or a more complex case *per se* hemodynamically, etc) and thus account for the observed association. Previous research has shown an association with the outcome and symptom duration, preoperative clinical status, angiographic success, age, and level of fistula (16-19).

This case-series suggests that surgery-first treatment strategy is more likely to yield a reasonably satisfactory outcome than embolization.

## References

[1] Mourier KL, Gelbert F, Rey A, George B, Reizine D, Merland JJ (1989). Spinal dural arteriovenous malformations with perimedullary drainage. Indications and results of surgery in 30 cases.. Acta Neurochir (Wien).

[2] Kuwayama N (2016). Epidemiologic survey of dural arteriovenous fistulas in Japan: clinical frequency and present status of treatment.. Acta Neurochir Suppl (Wien).

[3] Aminoff M, Logue V (1974). The prognosis of patients with spinal vascular malformations.. Brain.

[4] Steinmetz M, Chow M, Krishnaney A, Andrews-Hinders D, Benzel E, Masaryk T (2004). Outcome after the treatment of spinal dural arteriovenous fistulae: a contemporary single-institution series and meta-analysis.. Neurosurgery.

[5] Safaee M, Clark A, Burkhardt J, Winkler E, Lawton M (2018). Timing, severity of deficits, and clinical improvement after surgery for spinal dural arteriovenous fistulas.. J Neurosurg Spine.

[6] Abecassis I, Osbun J, Kim L. Classification and pathophysiology of spinal vascular malformations. In: Aminoff M, Boller F, Swaab D, eds. Handbook of clinical neurology. Amsterdam: Elsevier; 2003;135-143. 10.1016/B978-0-444-63640-9.00013-8 28552135

[7] Goyal A, Cesare J, Lu V, Alvi M, Kerezoudis P, Brinjikji W (2019). Outcomes following surgical versus endovascular treatment of spinal dural arteriovenous fistula: a systematic review and meta-analysis.. J Neurol Neurosurg Psychiatry.

[8] Fiaschi P, Prior A, Sbaffi P, Bizzi F, D’Andrea A, Cagetti B (2019). Spinal dural arteriovenous fistulas: clinical results and quality of life assessment with surgical treatment as a crucial therapy. the joint experience of two centers.. World Neurosurg.

[9] Hessler C, Regelsberger J, Grzyska U, Illies T, Zeumer H, Westphal M (2010). therapeutic clues in spinal dural arteriovenous fistulas – a 30 year experience of 156 cases.. Cent Eur Neurosurg.

[10] Ozpinar A, Weiner G, Ducruet A. Epidemiology, clinical presentation, diagnostic evaluation, and prognosis of spinal arteriovenous malformations. In: Aminoff M, Boller F, Swaab D, eds. Handbook of clinical neurology. Amsterdam: Elsevier; 2017;145-152. 10.1016/B978-0-444-63640-9.00014-X 28552136

[11] Jablawi F, Schubert G, Dafotakis M, Pons-Kühnemann J, Hans F, Mull M (2020). Long-term outcome of patients with spinal dural arteriovenous fistula: the dilemma of delayed diagnosis.. AJNR Am J Neuroradiol.

[12] Tsuruta W, Matsumaru Y, Iihara K, Satow T, Sakai N, Katsumata M (2019). Clinical characteristics and endovascular treatment for spinal dural arteriovenous fistula in Japan: Japanese Registry of Neuroendovascular Therapy 2 and 3.. Neurol Med Chir (Tokyo).

[13] Koyalmantham V, Kale S, Devarajan L, Phalak M, Chandra P, Suri A (2020). Patient outcomes following obliteration of spinal dural arteriovenous fistula and the role of indocyanine green angiography videoangiography (ICG-VA) during surgery.. Neurol India.

[14] Shin D, Park K, Ji G, Yi S, Ha Y, Park S (2015). The use of magnetic resonance imaging in predicting the clinical outcome of spinal arteriovenous fistula.. Yonsei Med J.

[15] Cho W, Kim K, Kwon O, Kim C, Kim J, Han M (2013). Clinical features and treatment outcomes of the spinal arteriovenous fistulas and malformations.. J Neurosurg Spine.

[16] Ma Y, Chen S, Peng C (2018). Clinical outcomes and prognostic factors in patients with spinal dural arteriovenous fistulas: a prospective cohort study in two Chinese centres.. BMJ Open.

[17] Ofran Y, Yovchev I, Hiller N, Cohen J, Rubin S, Schwartz I (2013). Correlation between time to diagnosis and rehabilitation outcomes in patients with spinal dural arteriovenous fistula.. J Spinal Cord Med.

[18] Nagata S, Morioka T, Natori Y, Matsukado K, Sasaki T, Yamada T (2006). Factors that affect the surgical outcomes of spinal dural arteriovenous fistulas.. Surg Neurol.

[19] Cenzato M, Debernardi A, Stefini R, D’Aliberti G, Piparo M, Talamonti G (2012). Spinal dural arteriovenous fistulas: outcome and prognostic factors.. Neurosurg Focus.

